# Breaking barriers: shaping global health futures with pilot and feasibility initiative

**DOI:** 10.1186/s40814-024-01522-3

**Published:** 2024-07-03

**Authors:** Ambreen Nizar, Magdalena Janus

**Affiliations:** 1 Department of Pediatrics and Child Health, The Aga Khan University, Karachi, Pakistan; 2 Offord Centre for Child Studies, Department of Psychiatry and Behavioural Neurosciences, McMaster University, Hamilton, ON Canada

## Abstract

In the dynamic landscape of global health, the journey from a new development to its implementation is often fraught with challenges. Yet, it is within the crucible of these challenges that ingenuity flourishes and barriers are transcended. It is with great anticipation and enthusiasm that we introduce our special series, “Breaking barriers: shaping global health futures with pilot and feasibility initiatives.” This series will delve into the evidence surrounding the challenges of conducting health-related studies across diverse regions of the world.

## Background

Pilot and/or feasibility investigations constitute a crucial phase in the course of any research study. The insights gained through both quantitative and qualitative methods are invaluable, and the lessons learned from this phase shape the methodological aspects of the main study. The feasibility investigations and pilot work serve as an important bridge between conceptualization and execution, mitigating uncertainties in conducting extensive epidemiological research. These preliminary research activities establish the feasibility of more extensive future research [1–4]. The modifications made as a result of these studies demonstrate a commitment to rigorous research practices and contribute to the overall quality of research. Moreover, the findings from feasibility studies serve as valuable evidence when researchers seek additional funding or grants to support their future work.

In our own work [5], we have highlighted some uncertainties that had to be addressed before we could proceed with a large-scale validation study after developing a new tool, the Global Scales for Early Development (GSED), to assess child development [6]. The feedback from cognitive interviews and focus group discussions during the feasibility study helped the study authors revise and reorder a few items, add another local language for a specific country, add more relevant topics in the training module, and replace some objects from the kit to make them more contextually relevant.

We hope that this series will embrace a full spectrum of experiences that will help researchers appreciate and overcome the inherent issues of global health research, including studies that made key adjustments that enhanced the main study, as well as those that have had to scale down or even halt their efforts due to feasibility constraints. These narratives of resilience in the face of adversity serve as poignant reminders of the challenges inherent in global health research while also inspiring early-career researchers to persevere in the pursuit of improvement and impact. We encourage study teams to share their responses to the fundamental feasibility question, “Can we do this?” by presenting findings from their feasibility studies. Feasibility studies may traverse different tracks, encountering challenges concerning contextual issues, cultural and linguistic acceptability of adapted tools, administrative and training challenges, and the safety of new interventions. Below we provide a brief overview of these challenges as an encouragement for submission of manuscripts addressing any or all of them or describing new ones.

### Cultural and contextual sensitivities

Assessing the acceptability of interventions across global study populations is critically important. Feasibility studies often employ qualitative methodologies such as focus group discussions to explore cultural nuances and contextual factors that may influence intervention uptake and effectiveness. An example is the use of paperless data collection, which can be a reason for concern for the source population in one country and completely acceptable in other countries. A recent feasibility study reported qualitative findings on the acceptability of an intervention video among Black Americans, informing participants about genetic testing for cancer, resulting in modification of the video in preparation for a future randomized clinical trial [7].

### Acceptability of interview or survey questions

Ensuring the clarity and comprehensibility of interview or survey questions, especially if they are adapted or translated, is crucial for obtaining accurate and meaningful data from study participants. Pilot and feasibility studies often employ cognitive testing methods to assess the understandability of interview questions across different linguistic and cultural contexts [8]. In addition, in some linguistic or cultural contexts, the response options also may need to be modified, for example to limit social bias, as exemplified in a study conducted in Indonesia [9]. Addressing issues such as inaccurate translation, complex sentence structures, or culturally insensitive terminology enhances the validity and reliability of the data collected during subsequent research phases.

### Administrative challenges

Feasibility studies are frequently conducted to identify logistical hurdles that impede data collection and intervention implementation. These challenges may include difficulties in accessing target populations, scheduling participant visits, or logistical constraints in data collection methods. For example, a study conducted to test mindfulness in children found that its limitation was the inability to determine whether mindfulness administration was even feasible [10]. Addressing these administrative challenges early during the feasibility phase allows for the development of robust strategies to optimize study implementation. Another example is a study conducted in Soweto, South Africa to test the feasibility of delivery of an intervention by community health workers (CHWs) [11]. It was found that CHWs were dissatisfied with their existing working conditions and low salaries, and were not ready to take on new tasks despite the intervention being well received by participants.

### Training challenges

Insights gained from feasibility studies can inform the refinement of training modules, allowing for the incorporation of real scenarios and challenges encountered during pilot testing. Strengthening training modules enhances the capacity of research teams to effectively execute larger-scale studies and mitigate potential implementation barriers. A study group conducted a trial to assess the feasibility and acceptability of training midwives to deliver a behavioral intervention to prevent obesity during pregnancy in four NHS trusts in Northeast England [12]. A mixed-methods approach to gathering information from midwives revealed that it can be training-intensive when implemented in a large number of facilities.

### Safety of the new intervention

Pilot and feasibility studies are crucial for assessing the safety and potential harm of interventions before larger-scale implementation, ensuring participant welfare and ethical conduct. For example, results from a pilot study demonstrated that the new model for oral anti-cancer medication care was safe and highly acceptable to patients, warranting a definitive trial in Ireland [13]. They are particularly needed to ensure that any potential unforeseen adverse effects or negative findings regarding the efficacy of new interventions are uncovered prior to clinical trials.

## Conclusion

The journey from posing the question “Can we do this?” [14] to providing an answer of “Yes, we can!” or “We can't do this!” is richly informative for researchers and stakeholders alike. However, the papers describing this process, especially when the outcome is negative, are rarely published. It is important to emphasize that feasibility studies do not assess intervention effectiveness or establish causal relationships. Thus, readers of feasibility studies should bear this distinction in mind while setting their feasibility objectives and interpreting the findings [3, 15]. Useful guidance for reporting and planning is found in several publications [1, 16] as well as three other special series within Pilot and Feasibility Studies that focus on issues related to Intervention Development [17], developing PROMS [18] and Implementation Science [19].

We express our profound gratitude to the authors who are contributing their work to this series and further extend our invitation to research groups to share their findings from feasibility studies, illuminating successful strategies and revised implementation plans. We also invite readers to embark on this journey with us, to engage deeply with the insights presented in this series, and to join us in shaping the future of global health. Together, let us break barriers, challenge conventions, and forge new paths toward a healthier, more equitable world.
